# Multimodal evidence and technology−enabled accommodations for autistic adults: an evidential framework for autism research and practice

**DOI:** 10.3389/fpsyt.2026.1852731

**Published:** 2026-06-04

**Authors:** David Ruttenberg

**Affiliations:** University College London, London, United Kingdom

**Keywords:** autism spectrum, camouflaging, gender diversity, motor ASD, neurodiversity, participatory research, sensory accommodations, wearable technologies

## Abstract

Autistic adults often face challenges to the legitimacy of their own neurology, in part because narrowly deficit-centric frameworks continue to privilege externally observed impairment over converging evidence from neuroscience, physiology, lived experience, and technology. This article proposes evidential pluralism — grounded in the Russo-Williamson thesis that causal inference in the health sciences requires the integration of difference-making and mechanistic evidence — as a framework for understanding autistic adulthood, extended here to encompass five distinct claim types: phenomenological, mechanistic, diagnostic, intervention, and ethical or policy. Drawing on converging but heterogeneous evidence from cognitive and sensory neuroscience, research on masking and mental health, gender and gender diversity, participatory methods, and wearable-technology studies, the paper considers how improved recognition of neurobiological diversity may have contributed to spectrum expansion, while acknowledging the parallel roles of diagnostic broadening, ascertainment effects, instrument drift, and sex and gender-related diagnostic bias. The paper explores how wearable and AI-enabled accommodation systems might, in principle, support autistic adults in real time and generate longitudinal physiological data, while emphasising the need for rigorous validation, independent replication, and robust privacy and data-governance safeguards. A dedicated section addresses the full heterogeneity of autistic adulthood, including adults with the highest support needs. The article closes by outlining implications for research design, clinical practice, and policy, framing accommodations as a matter of rights rather than discretionary privilege.

## Introduction: from narrowly deficit-centric to pluralistic frameworks

Across clinical consultations, institutional assessments, and policy deliberations, autistic adults can encounter challenges to the legitimacy of their own neurology, particularly when externally observable impairment is privileged over converging evidence from lived experience, neurobiology, and context (1–3). A late-diagnosed woman may be told she cannot be autistic because she communicates too effectively; a high-masking adult may be judged against the very compensatory performance that has obscured their support needs (4–6). These are structural consequences of epistemic frameworks that give disproportionate weight to narrow behavioural indicators while treating other forms of evidence as secondary (1, 2). Evidential pluralism does not minimise the reality of clinical burden. For many autistic individuals — particularly non-speaking, minimally verbal, and high-support-needs adults — motor impairment, autonomic dysregulation, epilepsy, self-injurious behaviour, sleep disruption, and gastrointestinal distress constitute genuine clinical needs requiring scientific investigation, measurement, and active support. Rights-based accommodation and evidence-based intervention are complementary rather than competing goals.

This article argues that addressing these challenges requires not simply better instruments within the existing framework, but a different framework entirely: evidential pluralism, the principled integration of neuroscientific, biological/physiological, behavioural, technological, and first-person forms of knowledge as mutually constraining evidential bases. The framework is grounded in Russo and Williamson’s (7) thesis that robust causal inference requires both difference-making and mechanistic evidence, extended here to five claim types suited to adult autism research. Howick et al. (8) demonstrate the limitations of mechanistic evidence alone — directly relevant to single-stream clinical approaches in autism. The discussion applies this framework across four domains: neuroscience and sensory processing, masking and mental health, gender and gender diversity, and participatory and technological approaches.

The argument operates at three levels: empirical review of findings from neuroscience, physiology, epidemiology, participatory research, and technology-enabled monitoring; conceptual interpretation of how these streams support an evidentially pluralistic approach; and normative claims about epistemic authority, unnecessary clinical gatekeeping, and accommodations as a matter of rights -– grounded in, but not directly inferred from, the empirical record (2, 9).

## Positionality statement

The argument advanced here is informed by a background in cognitive neuroscience, artificial intelligence, and ethics, and by sustained engagement with autistic people and families, including close family experience. That positionality informs the emphasis on accountability to autistic lived experience without substituting for empirical evidence; the technology section carries an explicit in-text conflict-of-interest declaration, uses conditional language throughout, and should be read as discussion of an emerging research domain rather than an endorsement of any product.

## Evidential pluralism in autism science

Calls for more evidence-based practice in autism often implicitly privilege some kinds of data over others: randomised trials over qualitative accounts, clinician observation over self-report, laboratory measures over naturalistic monitoring. Yet no single evidential stream is sufficient to ground robust causal or explanatory claims. Russo and Williamson (7) formalised this as the requirement that causal inference integrate both difference-making and mechanistic evidence. Howick et al. (8) demonstrate the specific limitations of mechanistic evidence without difference-making support — a finding that applies directly to single-stream clinical reasoning in autism.

In autism in adulthood, the relevant evidential domains are broader still, spanning cognitive and sensory neuroscience, behavioural and epidemiological studies, wearable-derived technological traces, and the phenomenological testimony of autistic people themselves (3, 10, 11). Historically, the field has privileged neuroscience to legitimise diagnostic categories, behavioural studies to define impairment thresholds, and clinician judgment to arbitrate self-report credibility (1, 2), while technology has typically been treated as an intervention delivery mechanism rather than an evidential resource (12).

Building on the Russo-Williamson framework, this article proposes an extension suited to the breadth of claims in adult autism research. Different claim types require different forms of evidence: phenomenological claims are addressed by first-person testimony and participatory methods; mechanistic claims require neuroimaging or electrophysiology; diagnostic claims require psychometric, developmental, and longitudinal evidence; intervention claims require controlled evaluation and independent replication; and ethical and policy claims require normative reasoning informed by but not derived from the empirical record (7). Motor function — including motor planning, movement variability, and motor constraints on communication and expressive behaviour — constitutes a further central evidential domain: observable social and communicative behaviour is frequently downstream of motor difficulty, and physiological data must be interpreted in light of motor state (13–15). Evidential pluralism is therefore a framework of integration and mutual constraint, not a rejection of standards.

## Neuroscience and sensory evidence across autistic adulthood

When diagnostic debates focus narrowly on observable behaviour, they risk mistaking historically contingent descriptions for underlying neurobiology. A pluralistic evidential approach asks instead what patterns emerge when evidence is considered across neuroimaging, electrophysiology, sensory neuroscience, and lived experience in autistic adults with diverse behavioural presentations, including those who mask extensively and those who do not (10, 11).

The neuroscience is strongly convergent, even if not uniform across every study. Reviews of resting-state functional connectivity, white matter organisation, and event-related potentials have reported atypical patterns across autistic populations, while emphasising heterogeneity in effect size and presentation (16–18). This does not imply a unitary neurobiological profile, but suggests that autistic presentations recognised across adulthood — including late-diagnosed and high-masking groups — are not exhausted by historically narrow behavioural prototypes (19, 20).

At the same time, rising prevalence cannot be attributed to neurobiological discovery alone: diagnostic broadening, changing ascertainment, evolving screening tools, and sex and gender-related under-identification have each contributed to who is recognised as autistic and when (21–24). Broad spectrum framings can also obscure clinically meaningful subgrouping if phenotypic, developmental, and support-needs differences are collapsed too quickly (25–27). Improved recognition of neurobiological diversity is therefore an important contributor to spectrum expansion, but not the only one.

The neurobiological basis for sensory processing differences is also well supported. Sensory hyper- and hyporeactivity are formally included in DSM-5, and sensory neuroscience has documented atypical multisensory integration, altered habituation, and differences in sensory gating among autistic people (11, 28) — differences with direct implications for attention, executive load, distress, and environmental accessibility (11).

## Masking, mental health, and adult outcomes

Masking and camouflaging behaviours are among the most consequential and contested features of autistic adulthood. From an evidential pluralism perspective, the question is not whether self-reports of exhaustion and identity disruption are sufficient on their own, but how they align with neurobiological, physiological, and epidemiological data on stress and mental health (6, 29, 30).

Scepticism about masking as a scientifically valid phenomenon is increasingly difficult to sustain. Studies using the CAT-Q and related methods have linked masking to depression, anxiety, suicidality, burnout, and diminished sense of self, especially among autistic women and other highly socially monitored groups (6, 29–31). Empirical support for its physiological costs comes from a co-twin control study showing that higher camouflaging predicts elevated long-term cortisol — consistent with chronic HPA-axis activation — after controlling for familial factors (32).

The evidential significance of masking lies in the convergence between self-report, physiological stress indices, and downstream mental health burden — strengthening the case for treating masking as a clinically significant feature of autistic adulthood rather than a purely subjective construct (29, 30).

## Gender and gender diversity in autistic adults

Among the many clinically significant dimensions of heterogeneity in autistic adulthood — including motor impairment, communication profile, intellectual disability, autonomic dysregulation, and support-needs level — gendered and gender-diverse patterns in diagnosis, presentation, and mental health have also received growing attention and warrant inclusion in any pluralistic framework (4–6). Delayed diagnosis among autistic women, together with higher rates of camouflaging and misrecognition, has been documented repeatedly (4–6). At the same time, framing the problem as a simple binary correction risks replacing one reductive template with another. The so-called female autism phenotype is not a single presentation that can be added to a male norm, but a heterogeneous cluster shaped by neurobiology, socialisation, identity formation, and environmental expectations (5, 6).

Beyond sex-based patterns, autistic people are disproportionately likely to identify as transgender, non-binary, or otherwise gender-diverse, a recurrent feature of autistic population studies (33–35). Sex, gender, and gender diversity are relevant dimensions of the heterogeneity that an adequate framework must address, alongside motor impairment, intellectual disability, and support-needs profile.

## Autistic epistemic authority and participatory methods

Evidential pluralism is also about rebalancing whose knowledge counts — treating autistic adults as epistemic authorities on their own experience and designing research that positions them as co-researchers and co-designers rather than passive subjects (1, 2, 9).

This is both a normative and a methodological claim. Respect for autistic epistemic authority follows from commitments to autonomy, justice, and disability inclusion, and is reinforced by evidence that outsider-only framings consistently miss the salience of masking, sensory distress, burnout, and accommodation barriers (2, 3). Treating autistic adults as epistemic authorities does not mean every first-person report settles contested empirical questions; it means such reports constitute primary data to which other evidential streams must remain accountable, and that research must commit not just to broader sampling but to participatory co-design (1, 9, 36).

## Evidential pluralism across the full spectrum: high-support-needs autistic adults

Evidential pluralism must address the full heterogeneity of autistic adulthood, including non-speaking and minimally verbal adults, those with intellectual disability, motor-planning differences, epilepsy, gastrointestinal burden, autonomic dysregulation, and complex support needs (27, 37) -– populations chronically underrepresented in adult samples (25, 38).

For these populations, epistemic authority cannot be operationalised through verbal self-report alone; AAC, multimodal observation, and validated proxy-supported preference elicitation are necessary to avoid conflating speech absence with cognitive or phenomenological absence (39–41). The presumption of competence must be operationalised methodologically — through AAC-supported co-design, autistic informants as co-interpreters, and physiological monitoring as a proxy for internal states — not merely asserted as a value (39). Motor speech impairment is a significant predictor of expressive language capacity in minimally verbal autistic individuals, and its presence shapes the interpretation of communicative behaviour and self-report capacity (42, 43). Motor noise — continuous micro-movement variability — constitutes a biologically meaningful signal rather than measurement artefact, and wearable systems sensitive to motor state offer a route to clinically meaningful longitudinal data in this population (44, 45).

This issue also intersects with motor differences and complex medical comorbidity. Behavioural and communicative regulation may be shaped by apraxia-like motor-planning problems, sleep dysregulation, gastrointestinal burden, autonomic dysregulation, and seizure-related factors beyond autism-specific mechanisms (27, 37). Wearable physiological monitoring holds particular promise for this population — providing continuous data on arousal and dysregulation for adults unable to verbally report internal states, with systematic review evidence supporting feasibility across autistic populations of varying verbal ability (46). A genuine evidential pluralism must expand both the evidence it accepts and the autistic lives its methods are designed to illuminate.

## Wearable technology and AI-enabled accommodations

Wearable and AI-enabled systems may serve at least four distinct functions: (1) real-time sensory and attentional accommodation; (2) continuous physiological and motor-state measurement, including detection of autonomic instability, motor dysregulation, seizure-related risk, sleep disruption, and pain-related distress; (3) intervention-response tracking and individualised outcome prediction; and (4) mechanistic discovery through longitudinal, ecologically valid data. These functions are not mutually exclusive, but distinguishing them is important for clarifying what forms of validation each requires (12). Properly designed, such systems may both support individuals in real time and generate longitudinal physiological and motor data about sensory, social, and cognitive burden that are otherwise difficult to capture in clinic-based assessments — particularly for non-speaking and high-support-needs adults.

Because the author holds patents on wearable sensory accommodation systems, the claims in this section must be read with that conflict of interest in mind. They are proposals grounded in early-stage evidence, not endorsements of any product or assertions of established clinical efficacy; independent replication and external validation are necessary conditions for any claim of clinical utility.

Wearable monitoring systems typically combine heart rate variability, galvanic skin response, accelerometry, and environmental signals (noise, light exposure); machine-learning models estimate individualised baselines and flag deviations indicative of stress or overload. Validity depends on real-world reliability, cross-profile generalisation, and correspondence between outputs and clinically meaningful states rather than artefact (12).

Current evidence is promising but limited. Systematic reviews confirm the feasibility of physiological stress sensing across autistic populations of varying verbal ability, though most included studies involve children or adolescents rather than adults (46). Youth-focused biosensor work has demonstrated proof-of-concept for short-term prediction of physiological escalation (47), and consumer-grade monitors show acceptable heart-rate-derived arousal accuracy in autistic children under controlled conditions (48). Early pilot work in autistic and ADHD adults, including trials of co-designed sensory-to-mental-health accommodation systems, suggests it is possible in principle to close this loop in adult populations (49). The adult−specific evidence base nonetheless remains considerably weaker on long−term clinical utility, real−world accommodation efficacy, and quality−of−life outcomes (12). False positives may generate intrusive alerts; false negatives may miss clinically significant overload; individual baseline variability is substantial. These remain open empirical questions requiring larger, adult−focused, independently conducted evaluation before any strong clinical utility claim can be made.

Continuous physiological monitoring raises non-trivial risks related to privacy, data ownership, secondary use, and algorithmic bias (50). For autistic people already subject to unnecessary clinical gatekeeping and surveillance, these risks are especially salient. These concerns are especially important for non-speaking autistic people identified as intellectually disabled, who have often been denied meaningful opportunities to demonstrate competence, agency, preference, and internal experience through conventional clinical or research methods. Any deployment must include strong protections for user control, data sovereignty, auditability, and strict limits on secondary use, with governance models explicitly shaped by autistic co-designers (49, 50).

The goal is not to replace clinical judgment with automated interpretation, but to explore whether first-person report, physiological monitoring, and environmental context can constrain one another in empirically testable, ethically accountable ways. Accommodation systems that are demonstrably beneficial — following rigorous, independent evaluation — and governed in ways accountable to autistic users should reduce unnecessary dependence on clinical gatekeeping; rights-based access and rigorous clinical evaluation are mutually reinforcing imperatives.

## Advancing research, practice, and policy in autistic adulthood

Narrowly deficit-centric or exclusively behaviour-first approaches to autistic adulthood are increasingly difficult to defend as sufficient on their own. This is not a claim that clinical assessment, symptom measurement, or evidence-based intervention are in tension with autistic dignity or autonomy — rigorous scientific investigation and rights-based accommodation are complementary rather than competing goals. An evidentially pluralistic framework — integrating neuroscientific, biological/physiological, behavioural, technological, and first-person knowledge, differentiated by claim type — offers a more clinically and ethically useful basis for action (2, 7).

### Research

Future studies should integrate multiple evidential streams — neuroimaging or electrophysiology, multiomic and other biological health metrics, participatory methods, ecological momentary assessment, and wearable sensing — in adult samples reflecting diversity of gender, race, communication profile, and support needs, and should actively include non-speaking and high-support-needs adults with outcome measures prioritising quality of life, autonomy, communication access, and sensory regulation (3, 9, 12, 27, 38).

### Practice

Clinicians should treat autistic adults’ self-reports as primary data while triangulating those reports with physiological, observational, and contextual evidence where appropriate (1, 2). Screening for masking-related exhaustion, burnout, suicidality, sensory overload, and communication barriers should become routine in adult care, especially for late-diagnosed, gender-diverse, and high-masking adults (6, 33). Practice frameworks must also be responsive to non-speaking and high-support-needs adults, for whom accommodation requires AAC-supported evidential pathways and adapted participatory assessment (38, 39). Screening should also include physical pain and underlying medical contributors, including GI distress, seizure activity, immune-related inflammation, sleep disruption, and other sources of physiological dysregulation that may present behaviourally.

### Policy

Policy frameworks should recognise underlying general health, sensory environments, communication barriers, and masking demands as structural determinants of wellbeing (2, 3), and should fund independent evaluation of participatory-designed accommodation technologies that remain under autistic users’ control and generate privacy-preserving evidence about which supports are effective and for whom (9, 50).

## Conclusion

Autism in adulthood cannot be adequately understood through any single evidential stream: behavioural observation, self-report, neuroscience, biology/general health data, epidemiology, and technology-derived data each capture something important, and each leaves something out. That gap is widest for non-speaking, minimally verbal, and high-support-needs autistic adults — those most systematically excluded from the research, clinical frameworks, and technologies meant to serve them. The task is to build frameworks in which multiple forms of evidence constrain one another while remaining ethically accountable to the full range of autistic lives they purport to explain. Evidential pluralism — grounded in the Russo-Williamson thesis, extended across five claim types, applied across the full heterogeneity of autistic adulthood — offers one principled route toward that goal.

## Data Availability

The original contributions presented in the study are included in the article/supplementary material. Further inquiries can be directed to the corresponding author.
